# Growth regulation of simian and human AIDS-related non-Hodgkin's lymphoma cell lines by TGF-β1 and IL-6

**DOI:** 10.1186/1471-2407-7-35

**Published:** 2007-02-26

**Authors:** Kristin R Ruff, Adriane Puetter, Laura S Levy

**Affiliations:** 1 Department of Microbiology & Immunology and Tulane Cancer Center, Tulane University Health Sciences Center, New Orleans, Louisiana, USA

## Abstract

**Background:**

AIDS-related non-Hodgkin's lymphoma (AIDS-NHL) is the second most frequent cancer associated with AIDS, and is a frequent cause of death in HIV-infected individuals. Experimental analysis of AIDS-NHL has been facilitated by the availability of an excellent animal model, i.e., simian Acquired Immunodeficiency Syndrome (SAIDS) in the rhesus macaque consequent to infection with simian immunodeficiency virus. A recent study of SAIDS-NHL demonstrated a lymphoma-derived cell line to be sensitive to the growth inhibitory effects of the ubiquitous cytokine, transforming growth factor-beta (TGF-beta). The authors concluded that TGF-beta acts as a negative growth regulator of the lymphoma-derived cell line and, potentially, as an inhibitory factor in the regulatory network of AIDS-related lymphomagenesis. The present study was conducted to assess whether other SAIDS-NHL and AIDS-NHL cell lines are similarly sensitive to the growth inhibitory effects of TGF-beta, and to test the hypothesis that interleukin-6 (IL-6) may represent a counteracting positive influence in their growth regulation.

**Methods:**

Growth stimulation or inhibition in response to cytokine treatment was quantified using trypan blue exclusion or colorimetric MTT assay. Intracellular flow cytometry was used to analyze the activation of signaling pathways and to examine the expression of anti-apoptotic proteins and distinguishing hallmarks of AIDS-NHL subclass. Apoptosis was quantified by flow cytometric analysis of cell populations with sub-G1 DNA content and by measuring activated caspase-3.

**Results:**

Results confirmed the sensitivity of LCL8664, an immunoblastic SAIDS-NHL cell line, to TGF-beta1-mediated growth inhibition, and further demonstrated the partial rescue by simultaneous treatment with IL-6. IL-6 was shown to activate STAT3, even in the presence of TGF-beta1, and thereby to activate proliferative and anti-apoptotic pathways. By comparison, human AIDS-NHL cell lines differed in their responsiveness to TGF-beta1 and IL-6. Analysis of a recently derived AIDS-NHL cell line, UMCL01-101, indicated that it represents immunoblastic AIDS-DLCBL. Like LCL-8664, UMCL01-101 was sensitive to TGF-beta1-mediated inhibition, rescued partially by IL-6, and demonstrated rapid STAT3 activation following IL-6 treatment even in the presence of TGF-beta1.

**Conclusion:**

These studies indicate that the sensitivity of immunoblastic AIDS- or SAIDS-DLBCL to TGF-beta1-mediated growth inhibition may be overcome through the stimulation of proliferative and anti-apoptotic signals by IL-6, particularly through the rapid activation of STAT3.

## Background

AIDS-related non-Hodgkin's lymphoma (AIDS-NHL) is the second most frequent cancer (following Kaposi's sarcoma) associated with AIDS, and is the cause of death in approximately 16% of HIV-infected individuals [1,2]. The lymphoma typically represents a clonal expansion of B-cell origin classified as high grade Burkitt lymphoma (BL) or diffuse large B-cell lymphoma (DLBCL) with centroblastic or immunoblastic features [3]. Epstein-Barr virus (EBV) infection of tumor cells is a frequent characteristic, occurring in 30% of AIDS-BL and up to 90% of AIDS-DLBCL depending on the histological subtype. Genetic lesions in c-*myc*, *bcl*-6 and/or *p53 *have also been implicated in some histologic subtypes of AIDS-NHL [4]. The lymphoma tends to occur as a late complication of AIDS in long-term survivors of the disease. Thus, as life expectancy of HIV-infected individuals has been prolonged by highly active antiretroviral therapy (HAART), it might be anticipated that the incidence of AIDS-NHL would increase. Indeed, recent reports differ as to the impact of HAART on the incidence of systemic AIDS-NHL, although improved response to standard therapy and increased survival have been widely reported [3,5]. Experimental analysis of AIDS-NHL pathogenesis has been facilitated by the availability of an excellent animal model for human AIDS, i.e., Simian Acquired Immunodeficiency Syndrome (SAIDS) in the rhesus macaque (*Macaca mulatta*) consequent to infection with simian immunodeficiency virus (SIV). SAIDS-related non-Hodgkin's lymphoma (SAIDS-NHL) occurs in the SIV-infected rhesus macaque with an incidence approximating human AIDS-NHL [6,7]. The lymphoma typically represents a clonal expansion of B-cell origin classified as DLBCL with immunoblastic features or, less frequently, as Burkitt's-like lymphoma [8]. Most cases of SAIDS-NHL are infected with rhesus lymphocryptovirus (RhLCV), a homologue of Epstein-Barr virus [9]. The tumors are widely disseminated in anatomic distribution involving extranodal sites, most frequently the gastrointestinal tract, the genitourinary tract and the heart [6,7].

Analysis of the disease in humans or simians implicates cytokine dysregulation as a fundamental influence in pathogenesis [10-12]. A role for interleukin-6 (IL-6), in particular, is supported by a large body of evidence [11-14]. IL-6 is a stimulator of B-cell proliferation and differentiation that is expressed in EBV-positive AIDS-BL and to high levels in AIDS-DLBCL of both centroblastic and immunoblastic types. Evidence indicates that the cytokine may be expressed from the tumor cells themselves or from tumor-infiltrating cells [11,13,15-17]. Tumor cells frequently express the receptor for IL-6, thus rendering them responsive to autocrine and/or paracrine IL-6-mediated stimulation [13,16]. The secretion of IL-6 is further significant in AIDS-NHL because it may render the tumor cells resistant to the cytotoxic effects of chemotherapeutic drugs and/or increase the side effects [10,18,19]. Growth inhibitory effects of cytokines have been implicated as well. In particular, a recent study using a SAIDS-NHL-derived cell line demonstrated the growth inhibitory effects of the ubiquitous cytokine, transforming growth factor-β (TGF-β). TGF-β was shown to reduce cell viability by 67% and to increase apoptosis by 69% (range, 33–111%). The authors concluded that TGF-β acts as a negative growth regulator of the lymphoma-derived cell line and, potentially, as an inhibitory factor in the regulatory network of AIDS-related lymphomagenesis [20]. These findings were unexpected, given that (1) many cancers, including leukemias and other tumors of hematopoietic origin, typically develop resistance to TGF-β as a mechanism for progression [21-23], and (2) the majority of SAIDS-related lymphomas are infected with RhLCV, whose human-specific counterpart, EBV, is known to impart resistance to TGF-β inhibition in human lymphoblastoid cell lines [24,25]. Indeed, the marked sensitivity to growth inhibition by TGF-β suggests that a counteracting positive influence may be operative in SAIDS-NHL.

The present study was conducted to assess whether other SAIDS-NHL and AIDS-NHL cell lines are similarly sensitive to the growth inhibitory effects of TGF-β, and to test the hypothesis that IL-6 may represent a counteracting positive influence in their growth regulation. The latter hypothesis was considered to be a reasonable possibility since IL-6 signaling is known to activate proliferative and survival pathways in target cells, including those mediated by signal transducer and activator of transcription-3 (STAT3), extracellular signal-regulated kinase 1/2 (ERK1/2), and the *bcl-2 *family of apoptotic regulators [26-28]. Further, a recent report using a cultured hepatoma cell line showed that IL-6 counteracted the growth inhibitory influence of TGF-β1, primarily through the activation of multiple anti-apoptotic signaling pathways [29]. The present results show that some lymphoma-derived cell lines of human and simian origin are sensitive to TGF-β-mediated growth inhibition, and that IL-6 signaling through multiple pathways moderates that inhibition. The results demonstrate the multiple mechanisms involved in the interaction between TGF-β and IL-6 signaling in AIDS- or SAIDS-NHL cell lines, and suggest a balance between positive and negative growth regulatory influences in this disease.

## Methods

### Cultured Cell lines and Reagents

LCL8664, originally derived from a case of SAIDS-NHL at the Tulane National Primate Research Center, was obtained from American Type Culture Collection (ATCC CRL-1805). LCL8664 cells were cultured in RPMI-1640 medium with 2 mM GlutaMAX-l, 10% heat-inactivated fetal bovine serum (FBS) and 10 μg/ml gentamicin sulfate (Invitrogen Corporation, Carlsbad, CA). The 2F7, BCBL-1 and UMCL01-101 cell lines were obtained from the AIDS and Cancer Specimen Resource at the University of California, San Francisco. 2F7 was maintained in RPMI-1640 medium with 2 mM GlutaMAX-I, 10% heat-inactivated FBS, 0.05 mM β-mercaptoethanol, 1 mM sodium pyruvate, and 10 μg/ml gentamicin. UMCL01-101 and BCBL-1 were maintained in the same medium as for 2F7 but without sodium pyruvate. Recombinant human IL-6 protein was obtained from Biosource International, Inc. (Camarillo, CA) and recombinant human TGF-β1 was obtained from R&D Systems, Inc. (Minneapolis, MN). LY294002 was obtained from Sigma-Aldrich Company (St. Louis, MO).

### DNA isolation and Southern blot analysis

Large molecular weight genomic DNA was isolated from lymphoma 8664 and from lymphoma-derived cell line LCL8664, and Southern blot analysis was performed, as previously described [30]. Organization of the immunoglobulin heavy chain (IgH) locus was examined using the lgHJ6 probe (DAKO Corporation, Carpinteria, CA).

### Cell proliferation assays

To quantify cell viability by trypan blue exclusion, live cells were enumerated after trypan blue staining using light microscopy. To quantify cell proliferation using the MIT tetrazolium dye reduction assay, cells were deposited in triplicate wells of a 96-well flat-bottom tissue culture plate at 2 × 10^5 ^cells per ml (LCL8664), 2.5 × 10^4 ^cells per ml (2F7), 1.75 × 10^5 ^cells per ml (BCBL-1) or 1 × 10^5 ^cells per ml (UMCL01-101). At regular intervals after the addition of cytokines, 10 μl of MTT reagent (R&D Systems, Minneapolis, MN) was added per 100 μl culture volume, and cells were incubated for 4 hours at 37°C. After 4 hours, 100 μl of detergent reagent (R&D Systems, Minneapolis, MN) was added to each well followed by incubation for 3 hours at 37°C. The absorbance at 595 nm (A_595_) of each sample well was measured using an automated plate reader. The entire experiment with each cell line was repeated at least twice. The results were analyzed statistically using one-way ANOVA with Bonferroni post-test with statistical significance considered as p < 0.05.

### Intracellular flow cytometry

For time course analyses of response to cytokines, cells were deposited in culture medium at 1 × 10^6 ^– 5 × 10^6 ^cells/ml, treated with IL-6 or TGF-β1 as indicated and collected at regular intervals thereafter. For studies involving a short time course of ≤ 60 minutes, cells were serum-starved for 24 hours in RPMI-1640 with 0.5% BSA before the addition of cytokines. Cells were fixed in 1% formaldehyde and incubated at room temperature for 10 minutes. Fixed cells were permeabilized while vortexing in ice-cold 90% methanol and incubated at 4°C for 30 minutes. Permeabilized cells were washed two times in a stain buffer of phosphate buffered saline (PBS) with 0.5% bovine serum albumin. For staining, 1 × 10^5 ^– 5 × 10^5 ^cells were incubated at room temperature with primary antibody for 1 hour and secondary antibody for 1 hour. Samples were then washed with PBS. Flow cytometry was performed using a Becton Dickinson FACSCalibur flow cytometer and interpreted with BD Bioscience CELLQuest Pro Software. Dead cells were excluded from analysis based on forward and side scatter pattern. Immunological reagents were Alexa Fluor 488-conjugated monoclonal antibodies directed against phosphorylated STAT3 (#557814), phosphorylated ERK1/2 (#612592) and phosphorylated p38MAPK (#612594), and phycoerythrin-conjugated monoclonal antibody against LMP-1 (# 550018) (BD Biosciences Pharmingen, San Diego, CA); polyclonal rabbit antibody against phosphorylated Akt (Cell Signaling Technology, Inc., Danvers, MA; #CS 9271s) with Alexa Fluor 488-conjugated goat anti-rabbit IgG (Cell Signaling Technology, Inc., Danvers, MA; #A11070); FITC-conjugated monoclonal antibody against Bcl-2 (Santa Cruz Biotechnology, Inc., Santa Cruz, CA; #sc-7382); and monoclonal antibody against Bcl-6 (Chemicon, Temecula, CA; # MAB4618) with Alexa Fluor 488-conjugated goat anti-mouse IgG (Cell Signaling Technology, Inc., Danvers, MA; #A21121). For analysis of sub-G1 DNA content, LCL8664 cells were treated with cytokine as indicated, fixed, permeabilized, and stained with 20 μg/ml propidium iodide (PI) in PBS containing 3.8 mM sodium citrate and 8 μg/ml RNase A at 37°C for 30 minutes. Cells were analyzed on a Becton Dickinson FACSCalibur flow cytometer and interpreted using BD Bioscience CELLQuest Pro Software and ModFit (Verity Software House Inc.). Each DNA content histogram was generated from at least 10,000 gated events. The experiment was repeated three times independently.

### Assay for Caspase-3 activity

A colorimetric assay for caspase-3 activity (CaspACE Assay System, Promega Corporation, Madison, WI) was used according to the manufacturer's instructions. LCL8664 cells were deposited at 1 × 10^6 ^cells/ml, treated with cytokines as indicated for 16 hours or 24 hours, harvested by centrifugation at 4°C, washed with ice-cold PBS and resuspended in Cell Lysis Buffer at a concentration of 10^8 ^cells/ml. Cells were lysed by repeated freeze-thaw cycles and incubated on ice for 15 minutes before centrifugation to collect the supernatant fraction. Caspase assays were performed in triplicate using 20 – 50 μg of whole cell lysate in a 100 μl volume in 96-well plates. Some samples were treated with the broad caspase inhibitor zVAD-fmk as a negative control. Plates were sealed, incubated at 37°C for 4 hours, and absorbance at 405 nm (A_405_) was measured using an automated plate reader. The results were analyzed statistically using one-way ANOVA with Bonferroni post-test with statistical significance considered as p < 0.05.

### Immunoblot analysis

Whole cell lysates were prepared by resuspending cells in RIPA buffer (50 mM Tris-HCI, pH 7.4, 150 mM NaCl, 1% Nonidet P-40, 0.5% sodium deoxycholate, 0.1% SDS) containing 100 μg/ml PMSF (phenylmethylsulfonyl fluoride; USB Corporation, Cleveland, OH), 1 mM sodium orthovanadate and 20 μg/ml aprotinin (Sigma-Aldrich Company (St. Louis, MO) on ice for 1 hour. Lysates were cleared by centrifugation for 15 minutes at 13000 rpm (4°C). The protein concentration in whole cell lysates was determined by the Bradford method (Bio-Rad, Hercules, CA). Protein samples (20 μg) were denatured in a solution containing 20% v/v glycerol, 1.25 M β-mercaptoethanol, 5% w/v SDS, 250 mM Tris-HCI, pH 6.7, 20% w/v bromophenol blue, boiled for 3 minutes and chilled on ice. Proteins were then separated by SDS-PAGE on a 10% acrylamide gel and transferred to a nitrocellulose membrane. Membranes were blocked from non-specific binding with 5% nonfat dried milk and PBS containing 0.1% tween-20 (PBST) for 1 hour at room temperature. Primary antibodies were directed against Mcl-1 (Santa Cruz Biotechnology, lnc., Santa Cruz, CA; #sc-819), LMP-1 and EBNA2 (DAKO Corporation, Carpinteria, CA; #M0897 and #M7004). Immune complexes were detected using horseradish peroxidase-conjugated secondary antibody (Southern Biotech, Birmingham, AL) and ECL Western Blotting Detection Reagents (Amersham Bioscience, Piscataway, NJ). Antibody to β-tubulin was used as a loading control.

## Results

**Figure 1:**
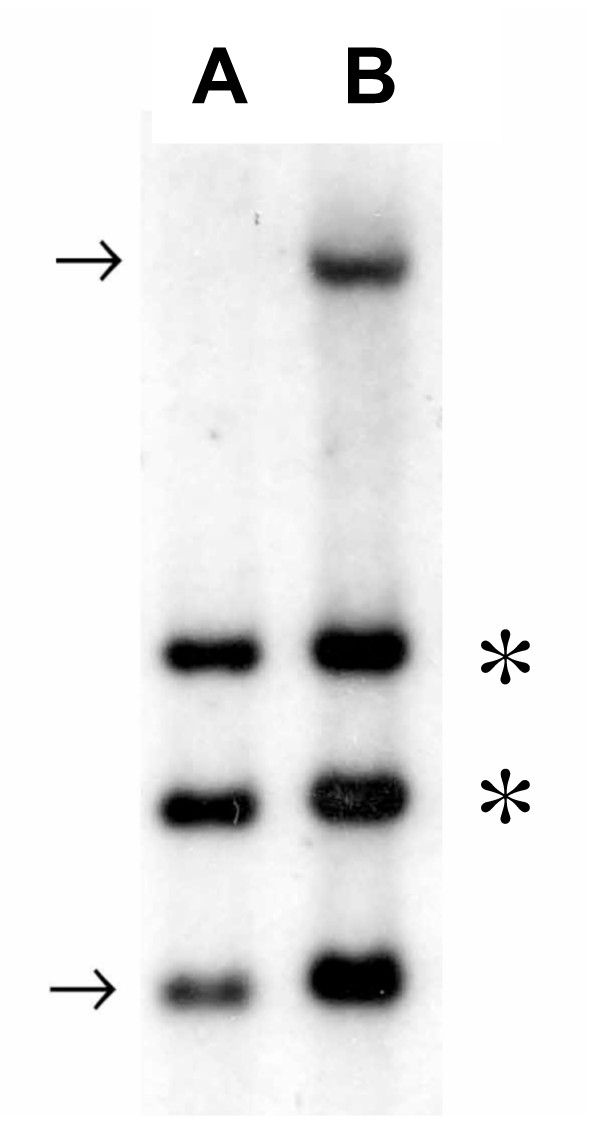
Southern blot analysis of the immunoglobulin heavy chain (IgH) locus in DNA from **(A) **LCL8664 cell line and **(B) **the original tumor mass from which LCL8664 cell line was derived. DNA (5 μg) was digested with *Sacl *and hybridized to a probe from the human IgH locus, 3' to the J_H _region. The arrows indicate the fragments generated from the locus in germline organization. The asterisks indicate the clonal rearrangement of the IgH locus. DNA from the tumor mass contains a large germline fragment not present in the cell line, indicative of contamination of the original necropsy sample with non-neoplastic tissue.

**Figure 2:**
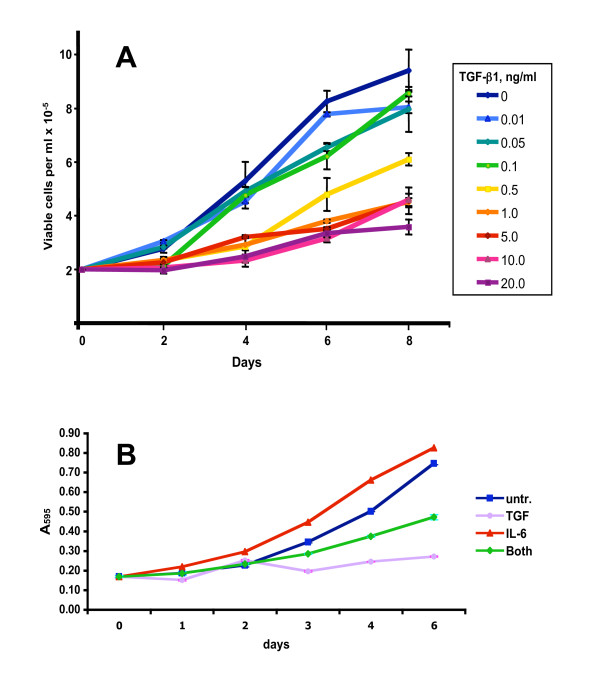
**(A)**. Dose-dependent growth inhibition of LCL8664 cells by TGF-β1. LCL8664 cells were cultured with TGF-β1 in varying concentrations from 0 ng/ml to 20 ng/ml. Viable cell counts were determined by trypan blue exclusion at regular intervals for 8 days. **(B)**. Positive and negative growth regulation of LCL8664 cells by TGF-β1 and IL-6. LCL8664 cells were cultured with TGF-β1 (1 ng/ml), IL-6 (20 ng/ml), both, or were untreated **(untr)**. Cell viability was quantified over 6 days of treatment using a tetrazolium dye reduction assay.

Based on the previous report of sensitivity of a single SAIDS-NHL-derived cell line to TGF-β1-mediated inhibition [20], an investigation was initiated to determine whether the finding could be recapitulated in LCL8664, a SAIDS-NHL-derived cell line from the Tulane National Primate Research Center characterized morphologically as immunoblastoid DLBCL [8]. LCL8664, like the original tumor, is of B-cell origin and is infected with rhesus lymphocryptovirus (RhLCV), the rhesus homologue of human EBV [7,9]. Consistent with its immunoblastic phenotype, LCL8664 cells express the virus-encoded oncogenes LMP-1 and EBNA-2 [31](Levy et al., unpublished observations). Southern blot analysis demonstrated identical immunoglobulin heavy chain gene rearrangements in LCL8664 and in the original tumor mass, thereby verifying that LCL8664 represents the predominant malignant clone (Figure 1). LCL8664 cells treated with human TGF-β1 in increasing amounts from 0.01 ng/ml to 20 ng/ml showed statistically significant inhibition of cell growth and viability as measured by trypan blue exclusion, beginning at two days of treatment with TGF-β1 at doses as low as 0.05 ng/ml. The growth-inhibitory effect on was maximal and indistinguishable at doses between 1.0 ng/ml and 20 ng/ml of TGF-β1 (Figure 2A). These results were confirmed using an alternative cell viability assay (MTT; data not shown). The effect of IL-6 on the growth of LCL8664 cells was then measured when IL-6 was present alone or in combination with TGF-β1. IL-6 significantly stimulated the proliferation of LCL8664 cells after two days of treatment. When IL-6 and TGF-β1 were added together, the inhibitory effect of TGF-β1 was modulated and cell proliferation was significantly rescued as compared to TGF-β1 alone (Figure 2B). The effect was blocked by specific antibody to IL-6 (data not shown).

**Figure 3:**
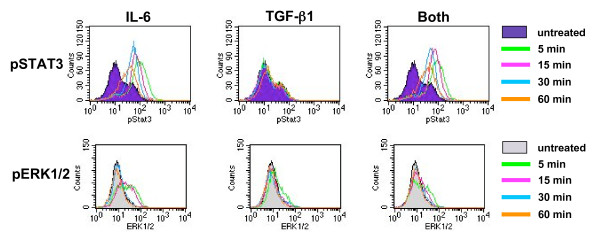
Activated (phosphorylated) STAT3 and ERK1/2 in LCL8664 cells treated with IL-6 and/or TGF-β1. LCL8664 cells were treated with IL-6 (20 ng/ml), TGF-β1 (1 ng/ml), both, or were untreated for time intervals between 0 – 60 minutes. Phosphorylated STAT3 **(pSTAT3) **and phosphorylated ERK1/2 **(pERK1/2) **were detected by intracellular flow cytometry.

Studies were then performed to determine whether IL-6 modulates the growth-inhibitory influence of TGF-β1 in LCL8664 cells through effects on cell proliferation, survival or both. To examine whether IL-6 activates STAT3 in SAIDS-NHL as it does in other target cells [26], LCL8664 cells were treated with IL-6, TGF-β1 or a combination of the two cytokines for time periods ranging from 0 to 60 minutes. Intracellular flow cytometry performed at regular intervals demonstrated the rapid activation of STAT3 within 5 minutes of IL-6 treatment. TGF-β1 treatment alone had no effect on STAT3 activation, nor did TGF-β1 treatment delay or diminish STAT3 activation when added together with IL-6. Thus, IL-6 activates the STAT3 pathway even in the presence of TGF-β1 (Figure 3). As IL-6 signaling also activates the primarily mitogenic Ras/Raf/ERK mitogen-activated protein kinase (MAPK) signaling pathway [26,28], similar intracellular flow cytometric analysis was performed to examine whether IL-6 activates this pathway in SAIDS-NHL. The results demonstrated rapid ERK1/2 phosphorylation within 5 minutes of IL-6 treatment, and ERK1/2 activation persisted for at least 60 minutes. TGF-β1 treatment alone had little or no effect on ERK1/2 activation, but the addition of TGF-β1 treatment blunted the activating effect of IL-6. Specifically, when both IL-6 and TGF-β1 were added together, ERK1/2 activation was observed with similar timing as with IL-6 alone but to a diminished extent. Thus, the presence of TGF-β1 reduces but does not block ERK1/2 activation in response to IL-6 (Figure 3). In contrast, p38MAPK was not activated in LCL8664 cells by any treatment tested (data not shown), consistent with the known activation of p38MAPK by environmental stress rather than by cytokine stimulation [10].

**Figure 4:**
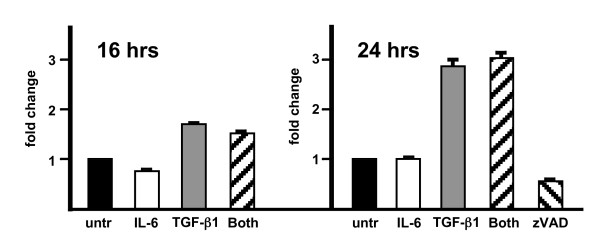
Caspase-3 activity in LCL8664 cells treated with IL-6 and/or TGF-β1. LCL8664 cells were treated with IL-6 (20 ng/ml), TGF-β1 (1 ng/ml), both, or were untreated **(untr) **for 16 or 24 hours. Parallel samples were treated with the broad caspase inhibitor, zVAD-fmk **(zVAD) **as a negative control. Caspase-3 activity was quantified in whole cell lysates using a colorimetric assay. The data are reported as fold-change in caspase-3 activity relative to untreated samples.

Since TGF-β induces apoptosis in several cell types [32-34] while the activation of STAT3 by IL-6 exerts a survival influence on target cells [27,28], the possibility was next considered that IL-6 signaling might rescue TGF-β1-mediated inhibition through an anti-apoptotic effect. LCL8664 cells treated with TGF-β1, IL-6 or a combination of the two cytokines for 24 hours were analyzed by flow cytometry to determine the proportion having a sub-G1 DNA content. In one representative analysis, the percentage of cells with sub-G1 DNA content increased from 5% in untreated cells to 12.5% after TGF-β1 treatment. Treatment of cells with both IL-6 and TGF-β1 decreased the percentage of cells with sub-G1 DNA content to 6.7%, thus indicating a partial protective effect of IL-6 even in the presence of TGF-β1. The induction of apoptosis following TGF-β1 treatment was further analyzed by measuring the expression of cleaved (active) caspase-3, an early event in the induction of programmed cell death. LCL8664 cells were treated with TGF-β1, IL-6 or a combination of both, and caspase-3 activity was quantified using a colorimetric assay at 16 hours and 24 hours thereafter. Consistent with the analysis of sub-G1 DNA content, significantly increased caspase-3 activity was evident by 16 hours of TGF-β1 treatment. A modest but statistically significant decrease in TGF-β1-induced caspase 3 activity was observed in the presence of IL-6 at 16 hours of treatment, but was no longer detectable after 24 hours (Figure 4).

**Figure 5:**
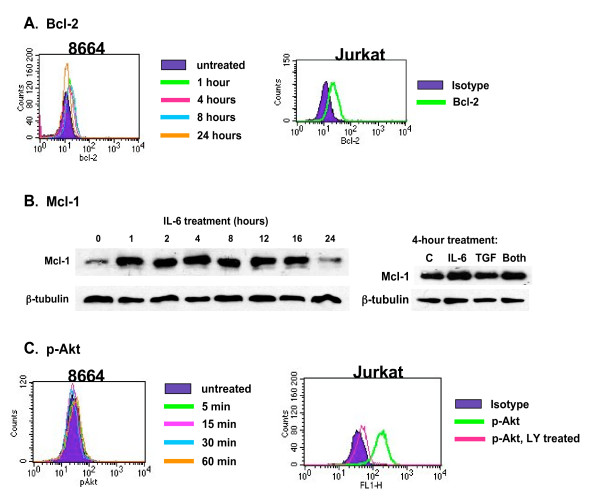
**(A)**.Bcl-2 expression in LCL8664 cells treated with IL-6. LCL8664 cells were treated with IL-6 (20 ng/ml), or were untreated **(untr)**, for time intervals between 1 – 24 hours. Bcl-2 expression was examined by intracellular flow cytometry. As a positive control, Bcl-2 expression in Jurkat cells was detected using the same methodology. **(B)**. Immunoblot analysis of Mcl-1 expression in LCL8664 cells treated with IL-6 and/or TGF-β1. In the left panel, LCL8664 cells were treated with IL-6 (20 ng/ml) for time intervals between 0 – 24 hours. In the right panel, LCL8664 cells were treated with IL-6 (20 ng/ml), TGF-β1 (1 ng/ml), both, or were untreated **(C) **for 4 hours. The expression of β-tubulin was examined as a loading control. **(C)**. Activated (phosphorylated) Akt expression in LCL8664 cells treated with IL-6. LCL8664 cells were treated with IL-6 (20 ng/ml), or were untreated **(untr)**, for time intervals between 0 – 60 minutes. Phosphorylated Akt **(p-Akt) **expression was examined by intracellular flow cytometry. As a positive control, p-Akt expression in Jurkat cells was detected using the same methodology, in the presence or absence of LY294002 (50 βM).

The anti-apoptotic signal stimulated by IL-6 is mediated ultimately through upregulated expression of Bcl-2 or its family members, Mcl-1 or Bcl-X_L _[27]. Intracellular flow cytometric analysis of LCL8664 cells treated with IL-6 for up to 24 hours demonstrated no effect on Bcl-2 expression, although the analysis clearly demonstrated constitutively elevated Bcl-2 expression in Jurkat T-cells as a positive control (Figure 5A). The expression of Bcl-2 family member, Mcl-1, was then measured by immunoblot in the absence of an antibody that functioned effectively for intracellular flow cytometry. In contrast to Bcl-2, Mcl-1 expression was significantly increased within 1 hour of IL-6 treatment, and upregulation persisted for 16 hours. Further, treatment with TGF-β1 did not diminish the ability of IL-6 to induce Mcl-1 expression (Figure 5B). Finally, considering that the phosphotidyl inositol 3-kinase/Akt signal pathway has been implicated in the regulation of Mcl-1 expression [35,36], LCL8664 cells treated with IL-6 for up to 60 minutes were examined by intracellular flow cytometry to quantify expression of phosphorylated (activated) Akt. The results demonstrated no effect of IL-6 treatment on Akt activation, although the analysis clearly demonstrated constitutively activated Akt in Jurkat T-cells, and its sensitivity to an inhibitor of phosphotidylinositol-3 phosphate kinase (LY294002), as a positive control (Figure 5C). Thus, IL-6 appears to exert an anti-apoptotic effect on LCL8664 cells through the induction of Mcl-1, although not through Akt or Bcl-2.

**Figure 6:**
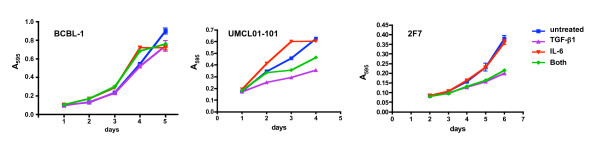
Positive and negative growth regulation of AIDS-NHL cell lines by TGF-β1 and IL-6. BCBL-1, UMCL01-101 and 2F7 cell lines were cultured with TGF-β1 (1 ng/ml), IL-6 (20 ng/ml), both, or were untreated. Cell viability was quantified at daily intervals thereafter using a tetrazolium dye reduction assay.

**Figure 7:**
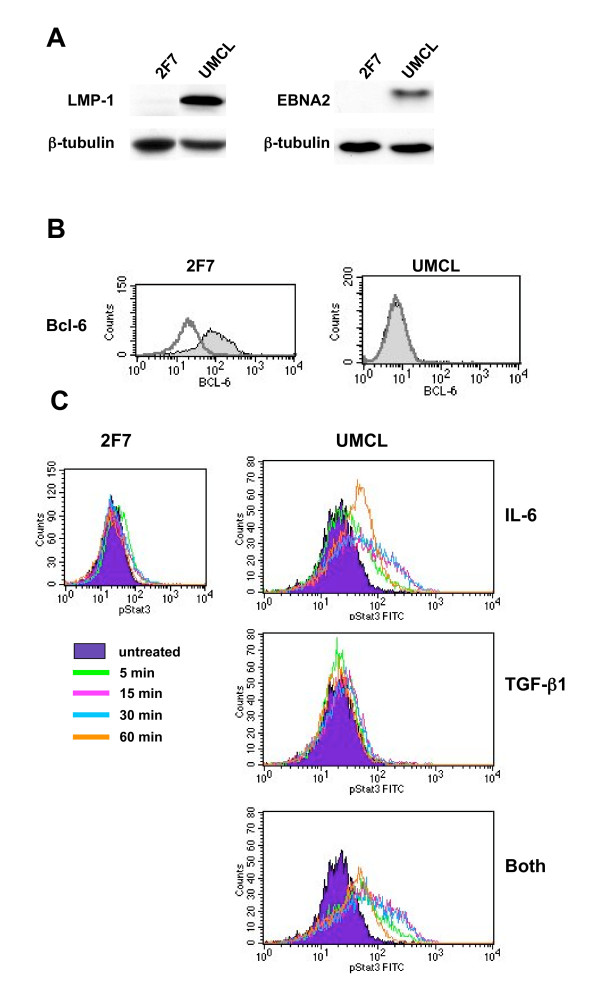
**(A)**. Immunoblot analysis of LMP-1 and EBNA2 expression in 2F7 and UMCL01-101 **(UMCL) **cell lines. The expression of β-tubulin was examined as a loading control. **(B)**. Bcl-6 expression in 2F7 and UMCL01-101 **(UMCL) **cell lines as examined by intracellular flow cytometry. The shaded and open histograms represent analysis with Bcl-6 antibody and isotype control antibody, respectively. **(C)**. Activated (phosphorylated) STAT3 in 2F7 and UMCL01-101 cell lines. 2F7 cells were treated with IL-6 (20 ng/ml), or were untreated, for time intervals between 0 – 60 minutes. UMCL01-101 cells **(UMCL) **were treated with IL-6 (20 ng/ml), TGF-β1 (1 ng/ml), both, or were untreated, for time intervals between 0 – 60 minutes. Phosphorylated STAT3 **(pSTAT3) **was detected by intracellular flow cytometry.

As LCL8664 and other SAIDS-NHL-derived cell lines are useful to the extent that they faithfully model AIDS-NHL, studies were next performed to establish which subtype(s) of AIDS-NHL is (are) effectively modeled by LCL8664 with respect to their response to IL-6 and TGF-β1. Three human AIDS-NHL-derived cell lines representing distinct histologic subtypes, i.e., 2F7, BCBL-1 and UMCL01-101, were treated with IL-6, TGF-β1, or a combination of both cytokines for 0 to 6 days, and growth was measured at regular intervals by MTT assay (Figure 6). BCBL-1 cell growth was modestly but significantly stimulated by IL-6, but was insensitive to treatment with TGF-β1. Since the growth of BCBL-1 cells was not inhibited by TGF-β1, this cell line was not further examined. Both 2F7 and UMCL01-101 showed marked sensitivity to TGF-β1-mediated growth inhibition, but only UMCL01-101 responded to IL-6 when added either alone or in combination with TGF-β1 (Figure 6). The findings suggest that LCL8664 may model the lymphoma subtype represented by UMCL01-101, and that LCL8664 and UMCL01-101 may share a similar molecular phenotype. To test this possibility, the expression of EBV-encoded oncogenes LMP-1 and EBNA2 was examined in 2F7 cells and UMCL01-101 cells by immunoblot analysis (Figure 7A). The results demonstrated that 2F7 cells, as is characteristic of AIDS-BL [11,37], do not generally express either LMP-1 or EBNA2. By contrast, UMCL01-101 cells express both LMP-1 and EBNA2 (Figure 7A), a pattern consistent with immunoblastic AIDS-DLBCL [3]. Further, intracellular flow cytometry demonstrated constitutive expression of Bcl-6 in 2F7 cells but not in UMCL01-101 (Figure 7B). This pattern of expression of LMP-1 and Bcl-6 in 2F7 and UMCL01-101 cells is considered a hallmark for the definition of AIDS-BL and immunoblastic AIDS-DLBCL, respectively [3]. Finally, studies were performed to determine whether IL-6 acts on human AIDS-NHL cells by STAT3 stimulation as it does in LCL8664. Consistent with the lack of responsiveness of 2F7 cells to IL-6 (Figure 6), no activation of STAT3 was detected after IL-6 treatment (Figure 7C). In contrast, the rapid activation of STAT3 was observed in UMCL01-101 cells within 5 minutes of IL-6 treatment. As was observed with LCL8664 cells (Figure 3), TGF-β1 treatment alone had no effect on STAT3 activation in UMCL01-101 cells, nor did TGF-β1 treatment delay or diminish STAT3 activation when added together with IL-6 (Figure 7C).

## Discussion

The present study was designed to examine the negative and positive growth regulatory influences of TGF-β1 and IL-6, respectively, on SAIDS- and AIDS-NHL cells. The LCL8664 cell line, although widely used as an experimental model [7,9,31], had not previously been documented to represent the original tumor mass. The demonstration by Southern blot analysis of identical immunoglobulin heavy chain gene rearrangements in the DNA of LCL8664 and the original tumor mass from which the cell line was derived confirmed their common origin. LCL8664 cells demonstrated a marked sensitivity to growth inhibition by TGF-β1 in a dose-dependent manner, an indication that lymphomas may be subject to significant growth suppressive influences *in vivo*. An intriguing recent report showed that IL-6 could counteract the growth inhibitory influence of TGF-β1 in a cultured hepatoma cell line. IL-6 was shown in that study to rescue cells from TGF-β1-mediated apoptosis by a mechanism dependent on activation of multiple anti-apoptotic signaling pathways [29]. Whether IL-6 can rescue B-lymphoid cells from TGF-β1-mediated growth inhibition had not previously been reported, although IL-6 is expressed in AIDS-NHL by the tumor cells themselves or by tumor-infiltrating cells [11,13,15-17]. Results of the present study showed that IL-6 stimulates the proliferation of LCL8664 cells, modulates the inhibitory effect of TGF-β1 and significantly rescues cell proliferation as compared to TGF-β1 alone. These findings suggest a mechanism by which growth suppressive influences in the lymphoma environment might be balanced.

The binding of IL-6 to its receptor initiates both proliferative and survival pathways in lymphoid malignancies, particularly through activation of the MAPK and STAT3 pathways, respectively [26,27,38]. IL-6 was shown to activate the STAT3 pathway in LCL8664 cells even in the presence of TGF-β1, the downstream effects of which may include the activation of both proliferation-associated and anti-apoptotic proteins that could counteract the growth-inhibitory effects of TGF-β1 [10,39,40]. Indeed, STAT3 activation has been previously implicated in the pathogenesis of AIDS-NHL [18], and in many other cancers including breast cancer, multiple myeloma, head and neck cancer, leukemias, lymphomas, lung cancer and prostate cancer [41-46]. Consistent with the known effect of IL-6 on the primarily mitogenic Ras/Raf/ERK MAPK signaling pathway [26,28], ERK1/2 was rapidly activated by IL-6 in LCL8664 cells, although to a lesser extent in the presence of TGF-β1. These findings suggest that a modest proliferative effect mediated by IL-6 through the ERK/MAPK pathway may contribute at least in part to the ability of IL-6 to rescue cells from TGF-β1-mediated inhibition. In addition to the potential proliferative influence, STAT3 activation exerts a survival influence on target cells. In multiple myeloma, for example, the inhibition of IL-6 receptor signaling through STAT3 was shown to induce apoptosis, an indication that IL-6-mediated STAT3 activation contributes to the pathogenesis of multiple myeloma by increasing the survival potential of tumor cells [41]. Analysis of sub-G1 DNA content and cleaved caspase-3 activity in LCL8664 cells indicated that TGF-β1 mediates growth inhibition through apoptotic induction, and that IL-6 treatment modestly but significantly diminishes the apoptotic effect. IL-6 appears to exert a survival influence on LCL8664 cells through induction of the Bcl-2 family member, Mcl-1, although not through Bcl-2 or Akt. Similarly, Mcl-1 has been identified as an essential survival protein in human myeloma cells [47,48] as well as in lymphoid malignancies and in normal lymphoid cells. Resistance to Fas-mediated apoptosis in large granular lymphocyte (LGL) leukemia has been attributed to STAT3 activation and increased Mcl-1 expression. The STAT3-mediated resistance to apoptosis of those cells was independent of Bcl-2 expression [49]. Further, a study of normal peripheral blood B-lymphocytes correlated steady levels of Mcl-1 with cell survival. Cells undergoing apoptosis demonstrated decreased Mcl-1 expression while Bcl-2 expression was unchanged. The application of survival stimuli such as interleukin-4 blocked the decrease in Mcl-1 expression [50]. Thus, our findings support a growing body of evidence implicating Mcl-1 as the critical survival protein in certain hematopoietic and lymphoid cells as well as in other cell types. Recent studies demonstrate that Mcl-1 may act in survival by sequestering Bim, thereby preventing a Bax-dependent apoptotic cascade mediated by free Bim, and consequently preserving mitochondrial membrane potential [51,52].

Analysis of three human AIDS-NHL-derived cell lines was undertaken to establish which subtype(s) might be faithfully modeled by the LCL8664 cell line with respect to the response to IL-6 and TGF-β1. 2F7 is an EBV-positive B-cell line established by single cell cloning from biopsy material of an AIDS-BL. Verification that 2F7 actually represents the original tumor clone was performed by Southern blot analysis to demonstrate identical immunoglobulin gene rearrangement [53]. BCBL-1 is a KSHV-positive, EBV-negative B-cell line derived from a body cavity-based AIDS-NHL [54,55]. UMCL01-101 was derived from an uncharacterized AIDS-NHL and was provided by the AIDS and Cancer Specimen Resource. The results demonstrated that AIDS-NHL cells are variably sensitive to TGF-β1-mediated growth inhibition and to IL-6 stimulation, an indication that the response to cytokines such as these may be a distinguishing characteristic of tumor cell phenotype. Of the cell lines examined, the response of UMCL01-101 most closely resembled that of LCL8664 in that the marked sensitivity to TGF-β1-mediated growth inhibition was rescued in part by treatment with IL-6. Further commonality of molecular phenotype was established through the demonstration that UMCL01-101 cells express EBV-encoded proteins LMP-1 and EBNA2, but do not express Bcl-6, a pattern definitive of immunoblastic AIDS-DLBCL [3]. Finally, as in LCL8664 cells, IL-6 was shown to activate the STAT3 pathway in UMCL01-101 even in the presence of TGF-β1. Thus, like in LCL8664 cells, IL-6 activation of the STAT3 pathway may account for the partial rescue of TGF-β1-mediated growth inhibition. Taken together, these studies indicate that UMCL01-101 likely represents an immunoblastic AIDS-DLBCL, and that it is effectively modeled by a SAIDS-NHL-derived cell line also characterized as immunoblastic. Moreover, the studies indicate that the sensitivity of immunoblastic AIDS-DLBCL to TGF-β1-mediated growth inhibition may be overeome through the stimulation of proliferative and anti-apoptotic signals by IL-6.

## Conclusion

Using an immunoblastic SAIDS-NHL cell line shown to represent the original tumor mass (Figure 1), initial studies confirmed a previous report [20] that SAIDS-NHL cells may be sensitive to TGF-β1-mediated growth inhibition in a dose-dependent manner (Figure 2A). The marked sensitivity to growth inhibition by TGF-β1 suggested that a counteracting positive influence may be operative in SAIDS-NHL. Based on a previous report that IL-6 counteracts the growth inhibitory influence of TGF-β1 in a cultured hepatoma cell line [29], the possible role of IL-6 was examined in the present system. Studies showed that IL-6 rescues LCL8664 cells, at least partially, from TGF-β1-mediated growth inhibition (Figure 2B), and that STAT3 activation occurs rapidly after IL-6 treatment even in the presence of TGF-β1 (Figure 3). The rapid activation of STAT3 signaling pathways may explain the ability of IL-6 to rescue cells from TGF-β1-mediated growth inhibition, since STAT3 is known to mediate proiliferative and anti-apoptotic signaling in normal and tumor cells [39,40]. The rapid activation of the ERK1/2 pathway by IL-6, somewhat blunted but not blocked by TGF-β1, indicates that proliferative stimulation contributes to the rescue of LCL8664 from TGF-β1-mediated inhibition. Flow cytometric cell cycle analysis and quantitation of caspase-3 activation indicated that TGF-β1 negatively regulates growth in LCL8664 at least in part by inducing apoptosis (Figure 4). IL-6 partially rescues LCL8664 cells from TGF-β1-mediated apoptosis, perhaps through activation of expression of the Bcl-2 family member, Mcl-1 (Figure 5). By comparison, human AIDS-NHL cell lines were observed to vary in their responsiveness to TGF-β1 and to IL-6. Of cell lines examined representing three different AIDS-NHL subtypes, only a recently derived, uncharacterized cell line designated UMCL01-101 demonstrated sensitivity to TGF-β1-mediated inhibition and partial rescue by IL-6 (Figure 6). Analysis of LMP-1 and Bcl-6 expression indicated that UMCL01-101, like LCL8664, represents immunoblastic AIDS-DLBCL (Figure 7A, 7B). Further analysis of the response to IL-6 demonstrated a rapid activation of STAT3, even in the presence of TGF-β1 (Figure 7C). Taken together, these findings indicate that immunoblastic AIDS-DLBCL remains sensitive to TGF-β1-mediated growth inhibition, and that the inhibition may be overeome through the stimulation of proliferative and anti-apoptotic signals by IL-6.

## Competing interests

The author(s) declare that they have no competing interests.

## Authors' contributions

KRR initially demonstrated the responsiveness of cell lines to TGF-β1 and IL-6. She developed and performed cell proliferation assays, caspase-3 assays and immunoblots. AP developed and performed intracellular flow cytometry. LSL directed the experimental design, implementation and interpretation of data. All authors read and approved the final manuscript.

## Pre-publication history

The pre-publication history for this paper can be accessed here:



## References

[B1] Goedert JJ (2000). The epidemiology of acquired immunodeficiency syndrome malignancies. Semin Oncol.

[B2] Levine AM, Seneviratne L, Espina BM, Wohl AR, Tulpule A, Nathwani BN, Gill PS (2000). Evolving characteristics of AIDS-related lymphoma. Blood.

[B3] Carbone A, Gloghini A (2005). AIDS-related lymphomas: from pathogenesis to pathology. Br J Haematol.

[B4] Gaidano G, Capello D, Carbone A (2000). The molecular basis of acquired immunodeficiency syndrome-related lymphomagenesis. Semin Oncol.

[B5] Diamond C, Taylor TH, Aboumrad T, Anton-Culver H (2000). Changes in acquired immunodeficiency syndrome-related non-Hodgkin lymphoma in the era of highly active antiretroviral therapy: incidence, presentation, treatment, and survival. Cancer.

[B6] Baskin GB, Murphey-Corb M, Watson EA, Martin LN (1988). Necropsy findings in rhesus monkeys experimentally infected with cultured simian immunodeficiency virus (SIV)/delta. Vet Pathol.

[B7] Habis A, Baskin GB, Murphey-Corb M, Levy LS (1999). Simian AIDS-associated lymphoma in rhesus and cynomolgus monkeys recapitulales the primary pathobiological features of AIDS-associated non-Hodgkin's lymphoma. AIDS Res Hum Retroviruses.

[B8] Baskin GB, Cremer KJ, Levy LS (2001). Comparative pathobiology of HIV- and SIV-associated lymphoma. AIDS Res Hum Retroviruses.

[B9] Habis A, Baskin G, Simpson L, Fortgang I, Murphey-Corb M, Levy LS (2000). Rhesus lymphocryptovirus infection during the progression of SAIDS and SAIDS-associated lymphoma in the rhesus macaque. AIDS Res Hum Retroviruses.

[B10] Jazirehi AR, Bonavida B (2005). Cellular and molecular signal transduction pathways modulated by rituximab (rituxan, anti-CD20 mAb) in non-Hodgkin's lymphoma: implications in chemosensitization and therapeutic intervention. Oncogene.

[B11] Knowles DM (2003). Etiology and pathogenesis of AIDS-related non-Hodgkin's lymphoma. Hematol Oncol Clin North Am.

[B12] Wood C, Harrington W (2005). AIDS and associated malignancies. Cell Res.

[B13] Fassone L, Gaidano G, Ariatti C, Vivenza D, Capello D, Gloghini A, Cilia AM, Buonaiuto D, Rossi D, Pastore C (2000). The role of cytokines in the pathogenesis and management of AIDS- related lymphomas. Leuk Lymphoma.

[B14] Hannig H, Matz-Rensing K, Kuhn EM, Stahl-Hennig C, Kaup FJ, Hunsmann G, Bodemer W (1997). Cytokine gene transcription in simian immunodeficiency virus and human immunodeficiency virus-associated non-Hodgkin lymphomas. AIDS Res Hum Retroviruses.

[B15] Emilie D, Coumbaras J, Raphael M, Devergne O, Delecluse HJ, Gisselbrecht C, Michiels JF, Van Damme J, Taga T, Kishimoto T (1992). lnterleukin-6 production in high-grade B lymphomas: correlation with the presence of malignant immunoblasts in acquired immunodeficiency syndrome and in human immunodeficiency virus-seronegative patients. Blood.

[B16] Marsh JW, Herndier B, Tsuzuki A, Ng VL, Shiramizu B, Abbey N, McGrath MS (1995). Cytokine expression in large cell lymphoma associated with acquired immunodeficiency syndrome. J Interferon Cytokine Res.

[B17] Pastore C, Gaidano G, Ghia P, Fassone L, Cilla AM, Gloghini A, Capello D, Buonaiuto D, Gonella S, Roncella S (1998). Patterns of cytokine expression in AIDS-related non-Hodgkin's lymphoma. BrJ Haematol.

[B18] Alas S, Bonavida B (2003). Inhibition of constitutive STAT3 activity sensitizes resistant non-Hodgkin's lymphoma and multiple myeloma to chemotherapeutic drug-mediated apoptosis. Clin Cancer Res.

[B19] Alas S, Emmanouilides C, Bonavida B (2001). Inhibition of interleukin 10 by rituximab results in down-regulation of bcl-2 and sensitization of B-cell non-Hodgkin's lymphoma to apoptosis. Clin Cancer Res.

[B20] Buske C, Hannig H, Schneider EM, Blaschke S, Hunsmann G, Bodemer W, Hiddemann W (1999). Transforming growth factor beta is a growth-inhibitory cytokine of B cell lymphoma in SIV-infected macaques. AIDS Res Hum Retroviruses.

[B21] DeCoteau JF, Knaus Pl, Yankelev H, Reis MD, Lowsky R, Lodish HF, Kadin ME (1997). Loss of functional cell surface transforming growth factor beta (TGF-beta) type 1 receptor correlatos with insensitivity to TGF-beta in chronic lymphocytic leukemia. Proc Natl Acad Sci USA.

[B22] Douglas RS, Capocasale RJ, Lamb RJ, Nowell PC, Moore JS (1997). Chronic lymphocytic leukemia B cells are resistant to the apoptotic effects of transforming growth factor-beta. Blood.

[B23] Kim SJ, Letterio J (2003). Transforming growth factor-beta signaling in normal and malignant hematopoiesis. Leukemia.

[B24] Fukuda M, Longnecker R (2004). Latent membrane protein 2A inhibits transforming growth factor-beta 1-induced apoptosis through the phosphatidylinositol 3-kinase/Akt pathway. J Virol.

[B25] Mori N, Morishita M, Tsukazaki T, Yamamoto N (2003). Repressionof Smad-dependent transforming growth factor-beta signaling by Epstein-Barr virus latent membrane protein 1 through nuclear factor-kappaB. Int J Cancer.

[B26] Heinrich PC, Behrmann l, Haan S, Hermanns HM, Muller-Newen G, Schaper F (2003). Principies of interleukin (IL)-6-type cytokine signalling and its regulation. Biochem J.

[B27] Hirano T, Ishihara K, Hibi M (2000). Roles of STAT3 in mediating the cell growth, differentiation and survival signals relayed through the IL-6 family of cytokine receptors. Oncogene.

[B28] Kamimura D, Ishihara K, Hirano T (2003). IL-6 signal transduction and its physiological roles: the signal orchestration model. Rev Physiol Biochem Pharmacol.

[B29] Chen RH, Chang MC, Su YH, Tsai YT, Kuo ML (1999). lnterleukin-6 inhibits transforming growth factor-beta-induced apoptosis through the phosphatidylinositol 3-kinase/Akt and signal transducers and activators of transcription 3 pathways. J Biol Chem.

[B30] Athas GB, Choi B, Prabhu S, Lobelle-Rich PA, Levy LS (1995). Genetic determinants of feline leukemia virus-induced multicentric lymphomas. Virology.

[B31] Blaschke S, Hannig H, Buske C, Kaup FJ, Hunsmann G, Bodemer W (2001). Expression of the simian Epstein-Barr virus-encoded latent membrane protein-1 in malignant lymphomas of SIV-infected rhesus macaques. J Med Virol.

[B32] Barna G, Sebestyen A, Chinopoulos CC, Nagy K, Mihalik R, Paku S, Kopper L (2002). TGF beta 1 kills lymphoma cells using mitochondrial apoptotic pathway with the help of caspase-8. Anticancer Res.

[B33] Chaouchi N, Arvanitakis L, Auffredou MT, Blanchard DA, Vazquez A, Sharma S (1995). Characterization of transforming growth factor-beta 1 induced apoptosis in normal human B cells and lymphoma B cell lines. Oncogene.

[B34] Inman GJ, Allday MJ (2000). Apoptosis induced by TGF-beta 1 in Burkitt's lymphoma cells is caspase 8 dependent but is death receptor independent. J Immunol.

[B35] Kuo ML, Chuang SE, Lin MT, Yang SY (2001). The involvement of Pl 3-K/Akt- dependent up-regulation of Mcl-1 in the prevention of apoptosis of HepSB cells by interleukin-6. Oncogene.

[B36] Wei LH, Kuo ML, Chen CA, Chou CH, Cheng WF, Chang MC, Su JL, Hsieh CY (2001). The anti-apoptotic role of interleukin-6 in human cervical cancer is mediated by up-regulation of Mcl-1 through a Pl 3-K/Akt pathway. Oncogene.

[B37] Bellan C, De Falco G, Lazzi S, Leoncini L (2003). Pathologie aspects of AIDS malignancies. Oncogene.

[B38] Fukada T, Hibi M, Yamanaka Y, Takahashi-Tezuka M, Fujitani Y, Yamaguchi T, Nakajima K, Hirano T (1996). Two signals are necessary for cell proliferation induced by a cytokine receptor gp130: involvement of STAT3 in anti-apoptosis. Immunity.

[B39] Bromberg J, Darnell JE (2000). The role of STATs in transcriptional control and their impact on cellular function. Oncogene.

[B40] Buettner R, Mora LB, Jove R (2002). Activated STAT signaling in human tumors provides novel molecular targets for therapeutic Intervention. Clin Cancer Res.

[B41] Catlett-Falcone R, Landowski TH, Oshiro MM, Turkson J, Levitzki A, Savino R, Ciliberto G, Moscinski L, Fernandez-Luna JL, Nunez G (1999). Constitutive activation of Stat3 signaling confers resistance to apoptosis in human U266 myeloma cells. Immunity.

[B42] Fernandas A, Hamburger AW, Gerwin BI (1999). ErbB-2 kinase is required for constitutive stat 3 activation in malignant human lung epithelial cells. Int J Cancer.

[B43] Gao B, Shen X, Kunos G, Meng Q, Goldberg ID, Rosen EM, Fan S (2001). Constitutive activation of JAK-STAT3 signaling by BRCA1 in human prostate cancer cells. FEBS Lett.

[B44] Garcia R, Bowman TL, Niu G, Yu H, Minton S, Muro-Cacho CA, Cox CE, Falcone R, Fairclough R, Parsons S (2001). Constitutive activation of StatS by the Src and JAK tyrosine kinases participales in growth regulation of human breast carcinoma cells. Oncogene.

[B45] Lin TS, Mahajan S, Frank DA (2000). STAT signaling in the pathogenesis and treatment of leukemias. Oncogene.

[B46] Weber-Nordt RM, Egen C, Wehinger J, Ludwig W, Gouilleux-Gruart V, Mertelsmann R, Finke J (1996). Constitutive activation of STAT proteins in primary lymphoid and myeloid leukemia cells and in Epstein-Barr virus (EBV)-related lymphoma cell lines. Blood.

[B47] Derenne S, Monia B, Dean NM, Taylor JK, Rapp MJ, Harousseau JL, Bataille R, Amiot M (2002). Antisense strategy shows that Mcl-1 rather than Bcl-2 or Bcl-x(L) is an essential survival protein of human myeloma cells. Blood.

[B48] Puthier D, Derenne S, Barille S, Moreau P, Harousseau JL, Bataille R, Amiot M (1999). Mcl-1 and Bcl-xL are co-regulated by IL-6 in human myeloma cells. Br J Haematol.

[B49] Epling-Burnette PK, Liu JH, Catlett-Falcone R, Turkson J, Oshiro M, Kothapalli R, Li Y, Wang JM, Yang-Yen HF, Karras J (2001). Inhibition of STAT3 signaling leads to apoptosis of leukemic large granular lymphocytes and decreased Mcl-1 expression. J Clin Invest.

[B50] Lomo J, Smeland EB, Krajewski S, Reed JC, Blomhoff HK (1996). Expression of the Bcl-2 homologue Mcl-1 correlates with survival of peripheral blood B lymphocytes. Cancer Res.

[B51] Han J, Goldstein LA, Gastman BR, Rabinowich H (2006). Interrelated roles for Mcl-1 and BIM in regulation of TRAIL-mediated mitochondrial apoptosis. J Biol Chem.

[B52] Weng C, Li Y, Xu D, Shi Y, Tang H (2005). Specific cleavage of Mcl-1 by caspase-3 in tumor necrosis factor-related apoptosis-inducing ligand (TRAIL)- induced apoptosis in Jurkat leukemia T cells. J Biol Chem.

[B53] Ng VL, Hurt MH, Fein CL, Khayam-Bashi F, Marsh J, Nunes WM, McPhaul LW, Feigal E, Nelson P, Herndier BG (1994). IgMs produced by two acquired immune deficiency syndrome lymphoma cell lines: Ig binding specificity and VH-gene putative somatic mutation analysis. Blood.

[B54] Komanduri KV, Luce JA, McGrath MS, Herndier BG, Ng VL (1996). The natural history and molecular heterogeneity of HIV-associated primary malignant lymphomatous effusions. J Acquir Immune Defic Syndr Hum Retrovirol.

[B55] Renne R, Blackbourn D, Whitby D, Levy J, Ganem D (1998). Limited transmission of Kapos i's sarcoma-associated herpesvirus in cultured cells. J Virol.

